# Tramadol regulates the activation of human platelets via Rac but not Rho/Rho-kinase

**DOI:** 10.1371/journal.pone.0279011

**Published:** 2023-01-13

**Authors:** Hiroki Iida, Takashi Onuma, Daiki Nakashima, Daisuke Mizutani, Takamitsu Hori, Kyohei Ueda, Tomoyuki Hioki, Woo Kim, Yukiko Enomoto, Tomoaki Doi, Rie Matsushima-Nishiwaki, Shinobu Yamaguchi, Junko Tachi, Kumiko Tanabe, Shinji Ogura, Toru Iwama, Osamu Kozawa, Haruhiko Tokuda

**Affiliations:** 1 Department of Anesthesiology and Pain Medicine, Gifu University Graduate School of Medicine, Gifu, Japan; 2 Department of Pharmacology, Gifu University Graduate School of Medicine, Gifu, Japan; 3 Department of Neurosurgery, Gifu University Graduate School of Medicine, Gifu, Japan; 4 Department of Metabolic Research, Research Institute, National Center for Geriatrics and Gerontology, Obu, Aichi, Japan; 5 Department of Dermatology, Kizawa Memorial Hospital, Minokamo, Japan; 6 Department of Emergency and Disaster Medicine, Gifu University Graduate School of Medicine, Gifu, Japan; 7 Department of Clinical Laboratory/Medical Genome Center, National Center for Geriatrics and Gerontology, Obu, Aichi, Japan; "INSERM", FRANCE

## Abstract

Tramadol is a useful analgesic which acts as a serotonin and noradrenaline reuptake inhibitor in addition to μ-opioid receptor agonist. Cytoplasmic serotonin modulates the small GTPase activity through serotonylation, which is closely related to the human platelet activation. We recently reported that the combination of subthreshold collagen and CXCL12 synergistically activates human platelets. We herein investigated the effect and the mechanism of tramadol on the synergistic effect. Tramadol attenuated the synergistically stimulated platelet aggregation (300 μM of tramadol, 64.3% decrease, p<0.05). Not morphine or reboxetine, but duloxetine, fluvoxamine and sertraline attenuated the synergistic effect of the combination on the platelet aggregation (30 μM of fluvoxamine, 67.3% decrease, p<0.05; 30 μM of sertraline, 67.8% decrease, p<0.05). The geranylgeranyltransferase inhibitor GGTI-286 attenuated the aggregation of synergistically stimulated platelet (50 μM of GGTI-286, 80.8% decrease, p<0.05), in which GTP-binding Rac was increased. The Rac1-GEF interaction inhibitor NSC23766 suppressed the platelet activation and the phosphorylation of p38 MAPK and HSP27 induced by the combination of collagen and CXCL12. Tramadol and fluvoxamine almost completely attenuated the levels of GTP-binding Rac and the phosphorylation of both p38 MAPK and HSP27 stimulated by the combination. Suppression of the platelet aggregation after the duloxetine administration was observed in 2 of 5 patients in pain clinic. These results suggest that tramadol negatively regulates the combination of subthreshold collagen and CXCL12-induced platelet activation via Rac upstream of p38 MAPK.

## Introduction

Tramadol is a widely used analgesic for acute and chronic pain, including postoperative pain, cancer pain, odontological pain, musculoskeletal pain and osteoarthritis pain in patients from children to elderly person [1, 2]. Pharmacologically, tramadol is recognized as a weak μ-opioid receptor (MOR) agonist which also acts as serotonin (5-hydroxy-tryptamine: 5-HT) and noradrenaline reuptake inhibitor (SNRI) [3]. The increase of bleeding risk is recognized as an undesirable side effect of selective serotonin reuptake inhibitor (SSRI), in which platelet dysfunctions at least in part are involved [4]. Actually, it has recently been reported in a large scaled case-control study that the treatment with tramadol is associated with an increased risk of bleeding peptic ulcer [5]. However, the contribution of the pharmacologic effects on platelet functions to the increased risk have not yet been evaluated [5]. A few studies in mammalian about the effects of tramadol on the platelet aggregation have been retrieved [6, 7], however, these findings have been quite controversial. Thus, the effect of tramadol on human platelet function has not yet been investigated.

Platelets play a critical role in hemostasis. Human platelets express serotonin transporter (SERT) on the surface of cell membrane [8]. Serotonin in plasma, secreted from intestinal cells, is taken into platelet cytoplasm through SERT [8], and then stored in dense granules through vesicular monoamine transporter (VMAT) [8, 9]. Once the vascular endothelium is damaged, tethering of platelets to the subendothelial tissue starts via receptors like Glycoprotein (GP) Ib/IX/V interacting with von Willebrand factor [10]. Then, the exposed subendothelial collagen triggers the activation and the accumulation of platelets via GPVI and integrin α2β1 on the platelets, which contributes to the platelet adhesion and subsequently leads to stabilized thrombus formation [11]. Activated platelets secrete several mediators including serotonin and ADP stored in the dense granules, platelet-derived growth factor-AB (PDGF-AB) stored in α-granules, and release soluble CD40 ligand (sCD40L) [10–12]. These mediators act to amplify further platelet activation in a case of positive feedback mechanism [10, 11]. Among them in particular, serotonin secreted from the dense granules acts via serotonin receptor (5-HT2A) expressed on the platelet surface to augment platelet activation [4, 9]. In addition to the function through 5-HT2A, serotonin retrieved through SERT is essential to sustain the platelet activation [13]. Transamidation of cytoplasmic serotonin to small GTPases such as Rho and Rac, called serotonylation, is proposed to play a role for platelet activation [9, 13]. The augmentation of cytoplasmic serotonin leads to increase serotonylation, which is closely related in fact to the pathogenesis of platelet hyperactivity in smoker [9]. Regarding the change in activated platelets, we previously reported that phosphorylated heat shock protein 27 (HSP27) is released from the activated platelet stimulated by collagen [14]. Evidences about extracellular HSP27 as an inflammation regulator has been accumulating [15, 16].

CXCL12 is a chemokine classified as CXC families, and functions via its specific G-protein-coupled receptors named CXCR4 and CXCR7 [17, 18]. As a chemokine, CXCL12 plays various roles in acute and chronic inflammation [17]. The serum levels of CXCL12 have been reported to be significantly high in patients with hyperlipidemia, diabetes and inflammatory diseases, risk factors for thrombotic diseases [19–23]. Mature human platelets express CXCR4 and CXCR7 on their surface and store CXCL12 in the α-granules [18]. It is known that activated platelets secrete CXCL12, and that the platelet-derived CXCL12 works to promote the repair of tissue and vascular injury [18]. CXCL12 by itself is a weak agonist for platelet activation, and reportedly enhances the responses of platelet to low-dose ADP, epinephrine, serotonine and collagen [24, 25]. Regarding the synergistic effect of CXCL12 and collagen on human platelet activation, we have recently reported that the low doses of collagen and CXCL12 in the range of subthreshold levels for the platelet aggregation synergistically act to induce platelet activation, and that GPVI and CXCR4 are the responsive receptors for collagen and CXCL12, respectively [26]. We also demonstrated that the activation of p38 mitogen-activated protein kinase (MAPK) but not p44/p42 MAPK is involved in the platelet activation synergistically stimulated by the combination of collagen and CXCL12, resulting in the PDGF-AB secretion, the sCD40L release, and the phosphorylated-HSP27 release [26]. Thus, the chemokine CXCL12 in the cooperation with collagen may play a pivotal role in the platelet activation in cases of inflammation with pain, the indication of analgesics such as tramadol. However, the precise regulatory mechanism underlying the synergistic effect of collagen and CXCL12 is not fully elucidated.

In the present study, we investigated the effect of tramadol on platelet activation by the combination of subthreshold levels of collagen and CXCL12 based on the pharmacological action to the target serotonylation of small GTPase. We show that tramadol negatively regulates the synergistic effect of subthreshold collagen and CXCL12 on platelet activation via the suppression of SERT to Rac at an upstream of p38 MAPK.

## Results

### Effect of tramadol on the simultaneous stimulation with collagen and CXCL12-induced human platelet aggregation; comparison with duloxetine, fluvoxamine, sertraline, morphine or reboxetine

**Fig 1:**
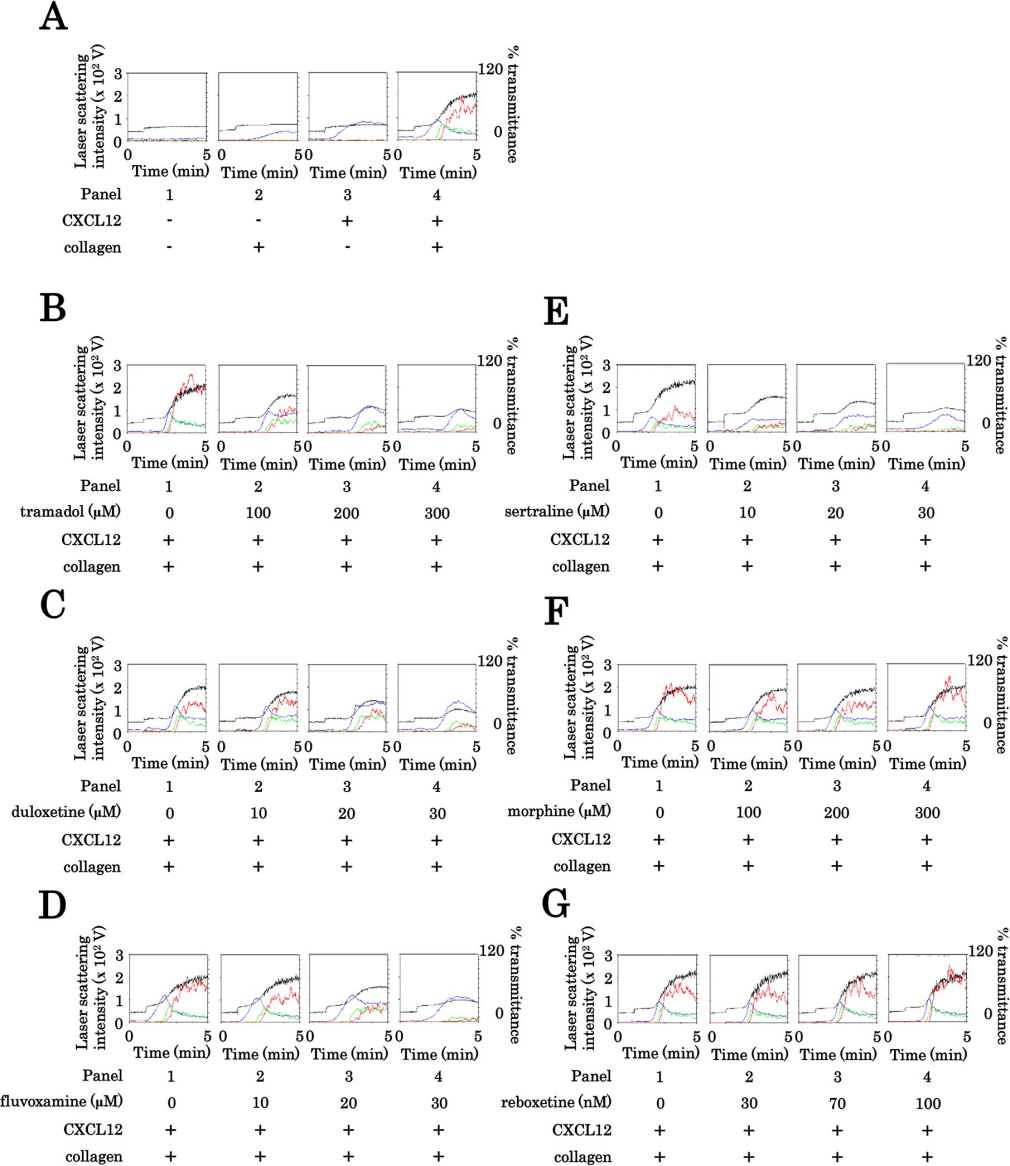
Effects of tramadol, duloxetine, fluvoxamine, sertraline, morphine or reboxetine on the platelet aggregation induced by the simultaneous stimulation with subthreshold collagen and CXCL12. (A) PRP was stimulated simultaneously by the combination of 0.2 μg/ml of collagen or vehicle and 10 ng/ml of CXCL12 or vehicle for 5 min. (B, C, D, E, F, G) PRP was pretreated with 0 μM, 100 μM, 200 μM or 300 μM of tramadol (B), 0 μM, 10 μM, 20 μM or 30 μM of duloxetine (C), 0 μM, 10 μM, 20 μM or 30 μM of fluvoxamine (D), 0 μM, 10 μM, 20 μM or 30 μM of sertraline (E), 0 μM, 100 μM, 200 μM or 300 μM of morphine (F), 0 nM, 30 nM, 70 nM or 100 nM of reboxetine (G) for 3 min, and then stimulated simultaneously by the combination of 0.075–0.45 μg/ml of collagen and 10 ng/ml of CXCL12 for 5 min. The representative results from 3 independent individuals are shown. The black line indicates the percentage of transmittance of each sample (isolated platelets recorded as 0%, and platelet-poor plasma recorded as 100%). The blue line indicates small aggregates (9–25 μm); green line, medium aggregates (25–50 μm); red line, large aggregates (50–70 μm).

At first, we confirmed that the subthreshold levels of collagen and CXCL12 synergistically elicited platelet aggregation (Fig 1A), as previously reported [26]. Thus, we investigated the effect of tramadol on the platelet aggregation induced by the combination of subthreshold collagen and CXCL12. Tramadol significantly attenuated the platelet aggregation dose-dependently up to 300 μM (Fig 1B). It is recognized that the peak concentration of tramadol in clinical use is 592 ± 178 ng/ml, which means about 3 μM [27]. The concentration of tramadol used here was much greater than the therapeutic range, however, it would be acceptable in the ex-vivo experiments to explore pharmacological effects in nova. Considering the pharmacological action of tramadol to inhibit serotonin reuptake [1], we next examined the effects of duloxetine, a SNRI [28], fluvoxamine, a SSRI [4], and sertraline, another SSRI [4] on the platelet aggregation synergistically stimulated by the combination of collagen and CXCL12 in the subthreshold levels. Duloxetine, fluvoxamine and sertraline attenuated the synergistically stimulated platelet aggregation in a dose-dependent manner up to 30 μM (Fig 1C–1E). We also examined the effects of morphine, a classical μ-opioid receptor agonist [29], and reboxetine, a noradrenaline reuptake inhibitor [30], on the platelet aggregation stimulated synergistically by the combination, however, neither morphine nor reboxetine affected the synergistically stimulated platelet aggregation up to 300 μM or 100 μM, respectively (Fig 1F and 1G). As well as tramadol described above, the therapeutic range of duloxetine, fluvoxamine, sertraline, morphine and reboxetine is recognized to be much higher than the dose in each which we used here [27]. However, it would be allowed to clarify pharmacological effects in ex-vivo experiments. Regarding the ratios of platelet aggregation size, tramadol, duloxetine, fluvoxamine and sertraline markedly decreased the prevalence of large aggregates (50–70 μm) but increased that of small aggregates (9–25 μm) (S1 Table). On the other hand, either morphine or reboxetine failed to decrease the prevalence of large aggregates (50–70 μm) or increase that of small aggregates (9–25 μm) (S1 Table).

### Effects of tramadol or fluvoxamine on the secretion of PDGF-AB and the release of sCD40L and phosphorylated-HSP27 from human platelets induced by the simultaneous stimulation with collagen and CXCL12

**Fig 2:**
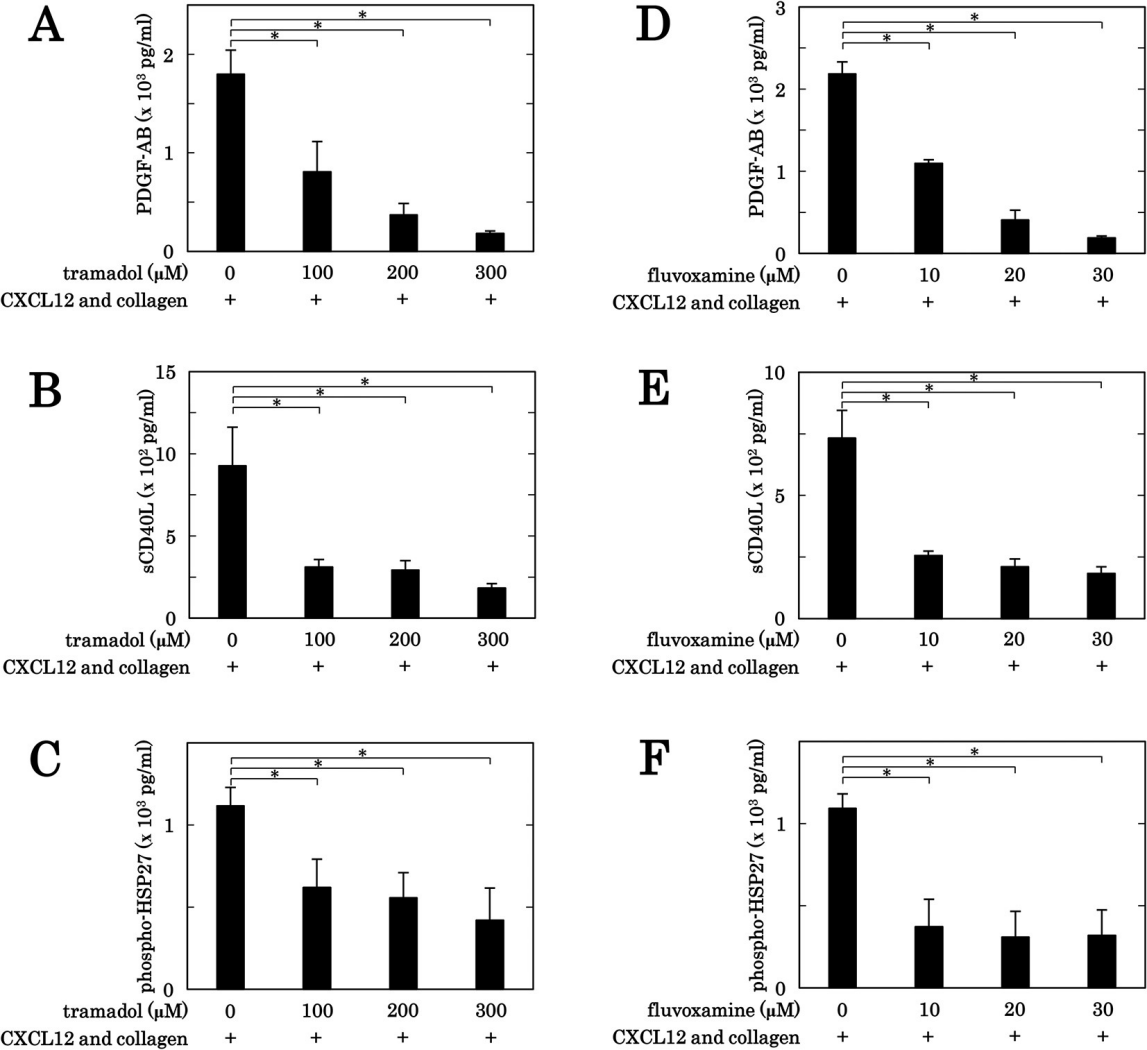
Effects of tramadol or fluvoxamine on the secretion of PDGF-AB, the release of sCD40L and the release of phosphorylated-HSP27 from human platelets induced by the simultaneous stimulation with subthreshold collagen and CXCL12. PRP was pretreated with 0 μM, 100 μM, 200 μM or 300 μM of tramadol (A, B, C), 0 μM, 10 μM, 20 μM or 30 μM of fluvoxamine (D, E, F) for 3 min, and then stimulated simultaneously by the combination of 0.075–0.45 μg/ml of collagen and 10 ng/ml of CXCL12 for 5 min (A, D) or 15 min (B, C, E, F). The reaction was terminated by the addition of ice-cold EDTA (10 mM) solution. The conditioned mixture was centrifuged at 10,000 × *g* for 2 min at 4°C, and the supernatant was collected. The levels of PDGF-AB (A, D), sCD40L (B, E) or phosphorylated-HSP27 (Ser-78) (C, F) in the supernatant was determined by ELISA. The results from 3 independent individuals are shown. Each value of PDGF-AB, sCD40L or phosphorylated-HSP27 (Ser-78) represents the mean ± SEM. *p<0.05, compared to the value of simultaneous stimulation with collagen and CXCL12.

We previously reported that the simultaneous stimulation with subthreshold collagen and CXCL12 induces not only the aggregation of platelet but also the secretion of PDGF-AB and the release of sCD40L and phosphorylated-HSP27 from platelets [26]. We next examined the effects of tramadol and fluvoxamine on the secretion of PDGF-AB, and the release of sCD40L and phosphorylated-HSP27 from human platelets stimulated synergistically by the combination of collagen and CXCL12 in the subthreshold levels. Tramadol suppressed the secretion of PDGF-AB (Fig 2A), the release of sCD40L (Fig 2B) and the release of phosphorylated-HSP27 (Fig 2C) from human platelets synergistically stimulated by the combination of collagen and CXCL12 in a dose-dependent manner in the range between 100 and 300 μM. Fluvoxamine also suppressed the secretion of PDGF-AB (Fig 2D), the release of sCD40L (Fig 2E) and the release of phosphorylated-HSP27 (Fig 2F) from human platelets stimulated synergistically by the combination in a dose-dependent manner in the range between 10 and 30 μM.

### Effects of FTase inhibitor III, GGTI-286, fasudil or Y27632 on the human platelet aggregation induced by simultaneous stimulation with collagen and CXCL12

**Fig 3:**
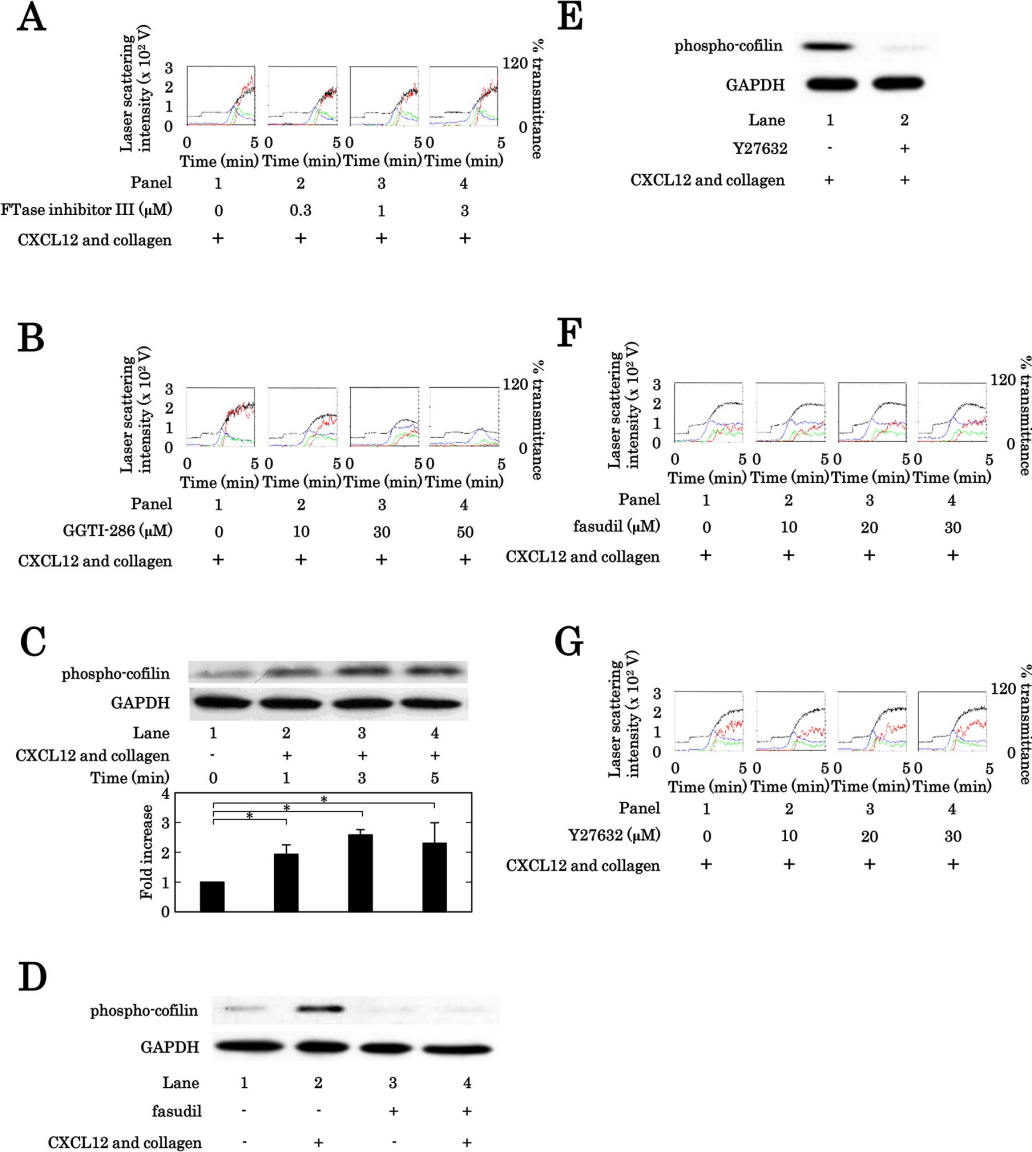
Effects of FTase inhibitor III, GGTI-286, fasudil and Y27632 on the platelet aggregation induced by simultaneous stimulation with subthreshold collagen and CXCL12. (A, B) PRP was pretreated with 0 μM, 0.3 μM, 1 μM or 3 μM of FTase inhibitor III (A), 0 μM, 10 μM, 30 μM or 50 μM of GGTI-286 (B) for 3 min, and then stimulated simultaneously by the combination of 0.3–0.35 μg/ml of collagen and 10 ng/ml of CXCL12 for 5 min. The representative results from 3 independent individuals are shown. The black line indicates the percentage of transmittance of each sample (isolated platelets recorded as 0%, and platelet-poor plasma recorded as 100%). The blue line indicates small aggregates (9–25 μm); green line, medium aggregates (25–50 μm); red line, large aggregates (50–70 μm). (C) PRP was simultaneously stimulated by 0.2 μg/ml of collagen and 10 ng/ml of CXCL12 for the indicated time, and the reaction was terminated by the addition of ice-cold EDTA (10 mM) solution. The levels of phosphorylated cofilin in the lysate of platelets were determined by the Western blot analysis using the antibodies against phospho-coffilin or GAPDH. The representative data from 3 independent experiments are shown. The histogram shows a quantitative representation of the combination-induced levels obtained from a densitometric analysis. The phosphorylation is expressed as the fold increase compared to the levels of lane 1. Each value represents the mean ± SEM of 3 times independent experiments. *p<0.05, compared to the value of lane 1. (D, E) PRP was pretreated for 3 min with 30 μM of fasudil (D), 30 μM of Y27632 (E) or vehicle, and then stimulated simultaneously by the combination of 0.15 μg/ml of collagen and 10 ng/ml of CXCL12 for 3 min. The levels of phosphorylated cofilin in the lysate of platelets were determined by the Western blot analysis as described above. The representative data of triplicate independent determinations are shown. (F, G) PRP was pretreated with 0 μM, 10 μM, 20 μM or 30 μM of fasudil (F), 0 μM, 10 μM, 20 μM or 30 μM of Y27632 (G) for 3 min, and then stimulated simultaneously by the combination of 0.075–0.45 μg/ml of collagen and 10 ng/ml of CXCL12 for 5 min. The representative results from 3 independent individuals are shown. The black line indicates the percentage of transmittance of each sample (isolated platelets recorded as 0%, and platelet-poor plasma recorded as 100%). The blue line indicates small aggregates (9–25 μm); green line, medium aggregates (25–50 μm); red line, large aggregates (50–70 μm).

From the results shown as Figs 1 and 2, it is likely that the inhibition of serotonin reuptake via SERT causes the suppression of the platelet activation induced by the combination of collagen and CXCL12 in their subthreshold levels. Regarding the function of SERT for platelet activation, it has been reported that serotonylation of small GTPases via serotonin uptake through SERT is involved in the regulation of platelet activation [9, 13]. It is well known that small GTPases such as Ras, Rho and Rac require prenylation to activate, and that two types of prenyltransferases, farnesyltransferase for Ras and geranylgeranyltransferase for Rho and Rac responsively catalyzes the prenylation [31]. We next examined the effects of FTase inhibitor III, an inhibitor of farnesylation [32], and GGTI-286, an inhibitor of geranylgeranylation [31], on the platelet aggregation induced by the combination of collagen and CXCL12 in their subthreshold levels. FTase inhibitor III up to 3 μM hardly affected the platelet aggregation synergistically stimulated by the combination (Fig 3A). Regarding the ratios of size, FTase inhibitor III failed to decrease the prevalence of large aggregates (50–70 μm) or increase that of small aggregates (9–25 μm) (S2 Table). On the other hand, GGTI-286 markedly suppressed the platelet aggregation stimulated synergistically by the combination dose dependently up to 50 μM (Fig 3B). Regarding the ratios of platelet aggregation size, GGTI-286 markedly caused the decrease of large aggregates (50–70 μm) and the increase of small aggregates (9–25 μm) (S2 Table). It is likely that not Ras but Rho or Rac is involved in the platelet aggregation simultaneously stimulated by the combination of subthreshold collagen and CXCL12.

It is generally recognized that cofilin, a small actin-activating protein, is a downstream target of Rho-kinase [33, 34]. The simultaneous stimulation by the combination of collagen (0.2 μg/ml) and CXCL12 (10 ng/ml) was confirmed to induce the phosphorylation of cofilin time dependently up to 5 min in human platelets (Fig 3C). Fasudil (30 μM) and Y27632 (30 μM), Rho-kinase inhibitors [35], were also confirmed to suppress the phosphorylation of cofilin induced by the simultaneous stimulation of the combination (Fig 3D and 3E). In order to explore the involvement of Rho-kinase in the synergistic effect of collagen and CXCL12 combination in their threshold levels on the platelet activation, we examined the effects of the Rho-kinase inhibitors on the platelet aggregation stimulated by the combination. However, neither fasudil nor Y27632 affected the platelet aggregation stimulated synergistically by the combination (Fig 3F and 3G). Regarding the ratios of platelet aggregation size, fasudil or Y27632 failed to change the prevalence of small aggregates (9–25 μm), medium aggregates (25–50 μm) and large aggregates (50–70 μm) (S2 Table). We also examined the effects of the Rho-kinase inhibitors on the secretion of PDGF-AB, the release of sCD40L and the release of phosphorylated-HSP27 (Ser-78) from platelets stimulated synergistically by the combination of collagen (0.2 μg/ml) and CXCL12 (10 ng/ml). Neither fasudil nor Y27632 affected the secretion of PDGF-AB (with 30 μM of fasudil, 2314.1 ± 142.1, control, 2330.2 ± 303.7 pg/ml, with 30 μM of Y27632, 2261.6 ± 143.5, control, 2082.8 ± 53.4 pg/ml), the release of sCD40L (with 30 μM of fasudil, 1056.7 ± 270.3, control, 881.7 ± 162.6 pg/ml, with 30 μM of Y27632, 557.3 ± 86.9, control, 584.6 ± 59.8 pg/ml) or the release of phosphorylated-HSP27 (Ser-78) (with 30 μM of fasudil, 475.3 ± 251.8, control, 612.0 ± 155.8 pg/ml, with 30 μM of Y27632, 929.7 ± 66.5, control, 1176.1 ± 147.9 pg/ml). It is unlikely that Rho is involved in the platelet activation induced by the simultaneous stimulation.

### Effects of NSC23766 on the human platelet activation and the phosphorylation of p38 MAPK and HSP27 in human platelets induced by the simultaneous stimulation with the combination of collagen and CXCL12

**Fig 4:**
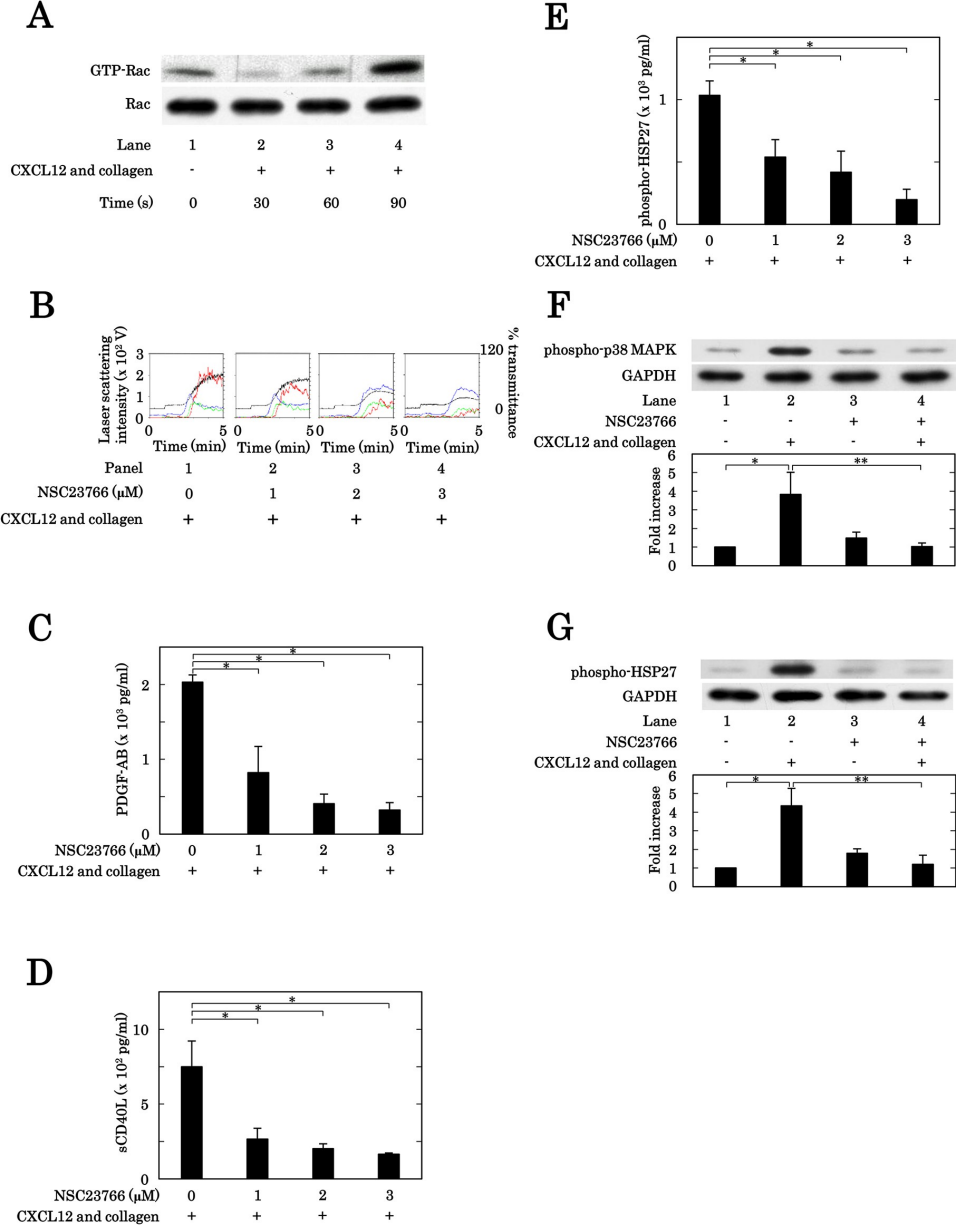
Effects of NSC23766 on the human platelet activation, the phosphorylation of p38 MAPK and the phosphorylation of HSP27 in human platelets induced by the simultaneous stimulation with collagen and CXCL12. (A) PRP was stimulated simultaneously by the combination of 0.2 μg/ml of collagen and 10 ng/ml of CXCL12 for the indicated periods. The reaction was terminated by the addition of ice-cold EDTA (10 mM) solution. The levels of GTP-binding Rac in the harvested protein of platelets were determined by the immunoprecipitated Western blot analysis. The representative data are shown. (B, C, D, E) PRP was pretreated for 3 min with 0 μM, 1 μM, 2 μM or 3 μM of NSC23766, and then stimulated simultaneously by the combination of 0.2–0.4 μg/ml of collagen and 10 ng/ml of CXCL12 for 5 min (B, C) or 15 min (D, E). The reaction was terminated by the addition of ice-cold EDTA (10 mM) solution. (B) The representative results of platelet aggregation from 3 independent individuals are shown. The black line indicates the percentage of transmittance of each sample (isolated platelets recorded as 0%, and platelet-poor plasma recorded as 100%). The blue line indicates small aggregates (9–25 μm); green line, medium aggregates (25–50 μm); red line, large aggregates (50–70 μm). (C, D, E) The conditioned mixture was centrifuged at 10,000 × *g* for 2 min at 4°C, and the supernatant was collected. The levels of PDGF-AB (C), sCD40L (D) or phosphorylated-HSP27 (Ser-78) (E) in the supernatant was determined by ELISA. The results from 3 independent individuals are shown. Each value represents the mean ± SEM. *p<0.05, compared to the value of simultaneous stimulation of collagen and CXCL12. (F, G) PRP was pretreated with 3 μM of NSC23766 or vehicle for 3 min, and then stimulated simultaneously by the combination of 0.2–0.4 μg/ml of collagen and 10 ng/ml of CXCL12 for 180 seconds. The reaction was terminated by the addition of ice-cold EDTA (10 mM) solution. and the levels of phosphorylated p38 MAPK (F) or phosphorylated HSP27 (G) in the lysate of platelets were determined by the Western blot analysis using the antibodies against phospho-p38 MAPK (F), against phospho-HSP27 (G) or against GAPDH. Each histogram shows a quantitative representation of the stimulation with collagen and CXCL12-induced levels obtained from a densitometric analysis. The density levels are expressed as the fold increase compared to the levels of vehicle, presented as lane 1. Each value represents the mean ± SEM of 3 times independent experiments. *p<0.05, compared to the value of vehicle. **p<0.05, compared to the value of simultaneous stimulation of collagen and CXCL12.

We next investigated the involvement of Rac in the human platelet activation simultaneously stimulated with the combination of collagen and CXCL12. We first confirmed that the increase of GTP-binding Rac levels was induced by the simultaneous stimulation with 0.2 μg/ml of collagen and 10 ng/ml of CXCL12 time-dependently up to 90 min in human platelets (Fig 4A). Thus, we examined the effects of NSC23766, a selective inhibitor of Rac1-GEF interaction [36], on the platelet aggregation stimulated synergistically by the combination of collagen and CXCL12. NSC23766 markedly suppressed the platelet aggregation stimulated synergistically by the combination (Fig 4B). Regarding the ratios of platelet aggregation size, NSC23766 markedly decreased the prevalence of large aggregates (50–70 μm) but increased that of small aggregates (9–25 μm) (S2 Table). We also examined the effects of NSC23766 on the secretion of PDGF-AB, the release of sCD40L and the release of phosphorylated-HSP27 (Ser-78) from human platelets simultaneously stimulated by the combination of subthreshold collagen and CXCL12. NSC23766 significantly reduced the secretion of PDGF-AB (Fig 4C), the release of sCD40L (Fig 4D) and the release of phosphorylated-HSP27 (Ser-78) (Fig 4E) from the human platelets synergistically stimulated by the combination in a dose-dependent manner up to 3 μM. It is likely that neither Ras nor Rho but Rac is involved in the platelet activation induced by the simultaneous stimulation.

In the proceeding study, we have reported that the simultaneous stimulation with the combination of collagen and CXCL12 in their subthreshold levels for platelet aggregation induces an activation of p38 MAPK and subsequent HSP27 phosphorylation resulting in the release of phosphorylated HSP27 from human platelets [26]. We then examined the effect of NSC23766 on the phosphorylation of p38 MAPK and that of HSP27 (Ser-78) stimulated simultaneously by the combination of collagen (0.2–0.4 μg/ml) and CXCL12 (10 ng/ml) in human platelets. NSC23766 (3 μM) significantly suppressed the phosphorylation levels of p38 MAPK in the platelets stimulated synergistically by the combination (Fig 4F). The phosphorylation of HSP27 (Ser-78) induced by the simultaneous stimulation by the combination was also inhibited by NSC23766 (3 μM) (Fig 4G).

### Effects of tramadol or fluvoxamine on the activation of Rac, and the phosphorylation of p38 MAPK and HSP27 in human platelets induced by the simultaneous stimulation with collagen and CXCL12

**Fig 5:**
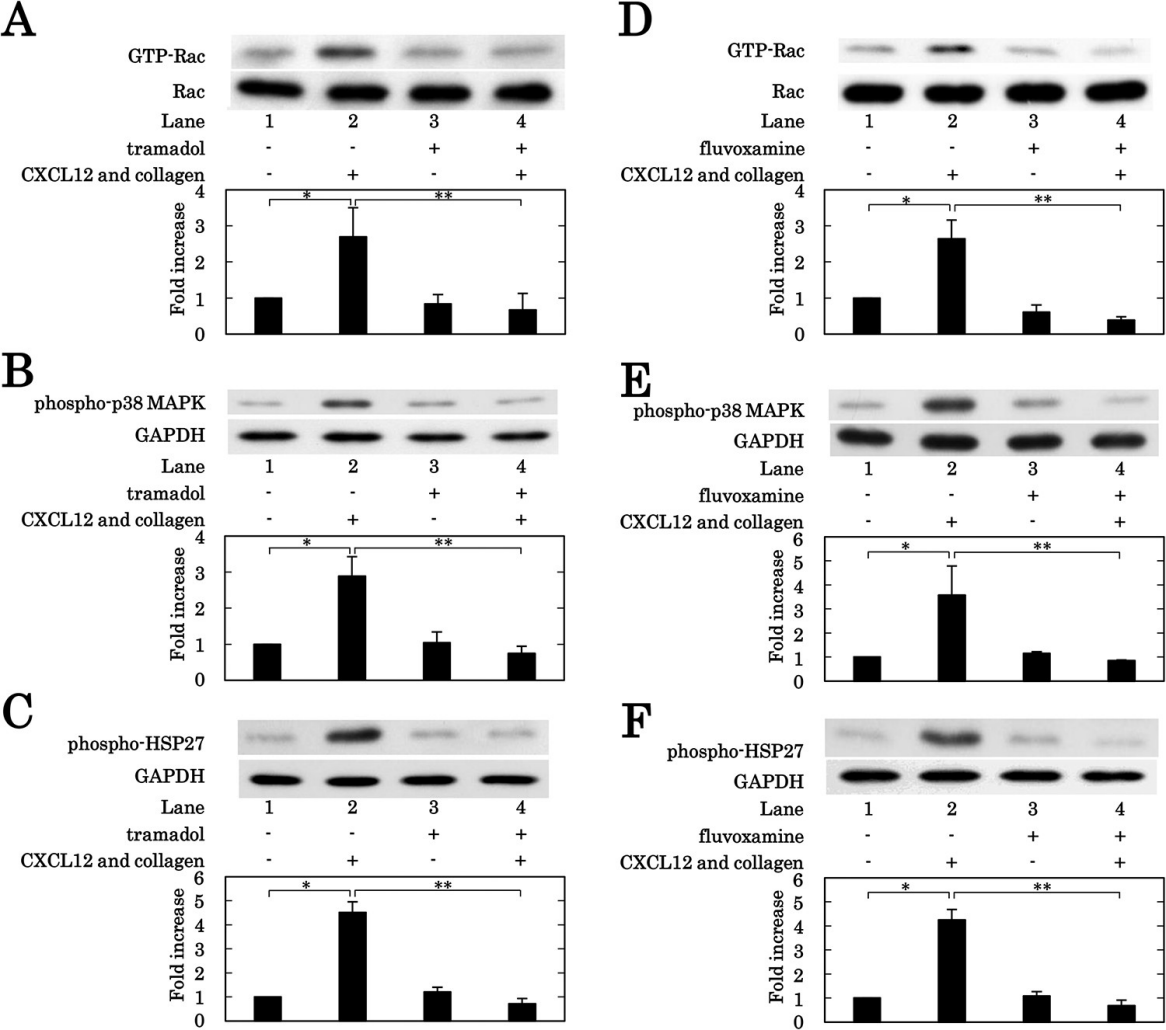
Effects of tramadol or fluvoxamine on the levels of GTP-binding Rac, the phosphorylation of p38 MAPK and the phosphorylation of HSP27 in human platelets induced by simultaneous stimulation with collagen and CXCL12. PRP was pretreated with 300 μM of tramadol (A, B, C), 30 μM of fluvoxamine (D, E, F) or vehicle for 3 min, and then simultaneously stimulated by 0.075–0.45 μg/ml of collagen and 10 ng/ml of CXCL12 for 90 seconds (A, D) or 180 seconds (B, C, E, F). The reaction was terminated by the addition of ice-cold EDTA (10 mM) solution. (A, D) The protein extracts were harvested as described in Materials and methods, and then GTP-binding Rac was immunoprecipitated using the Rac1 activation assay kit. The immunoprecipitated GTP-binding Rac and pre-immunoprecipitated lysates (Rac) were subjected to Western blot analysis using antibodies against Rac. (B, C, E, F) The levels of phosphorylated p38 MAPK or phosphorylated HSP27 in the cell lysate of platelets were determined by the Western blot analysis using anti-phospho-p38 MAPK antibodies (B, E), anti-phospho-HSP27 (Ser-78) antibodies (C, F) or anti-GAPDH antibodies. Each histogram shows a quantitative representation of the stimulation with collagen and CXCL12-induced levels obtained from a densitometric analysis. The density levels are expressed as the fold increase compared to the levels of vehicle, presented as lane 1. Each value represents the mean ± SEM of 3 times independent experiments. *p<0.05, compared to the value of vehicle. **p<0.05, compared to the value of simultaneous stimulation of collagen and CXCL12.

Considering the results presented above, it is likely that tramadol suppresses the platelet activation stimulated synergistically by the combination of subthreshold collagen and CXCL12 via inhibition of the serotonin uptake through SERT, and that the small GTPase Rac among serotonylation targets is involved at an upstream of p38 MAPK in the platelet activation. Thus, we further examined the effect of tramadol on the activation of Rac induced by the simultaneous stimulation with the combination of collagen and CXCL12 in human platelets. Tramadol markedly reduced the levels of GTP-binding Rac increased by the simultaneous stimulation with the combination (Fig 5A). Next, we examined the effect of tramadol on the phosphorylation of p38 MAPK induced by the simultaneous stimulation with the combination of collagen and CXCL12 in human platelets. Tramadol significantly suppressed the levels of p38 MAPK phosphorylation upregulated by the simultaneous stimulation (Fig 5B). We also examined the effect of tramadol on the phosphorylation of HSP27 (Ser-78) induced by the simultaneous stimulation with the combination of collagen and CXCL12 in human platelets. Tramadol significantly downregulated the levels of HSP27 (Ser-78) phosphorylation induced by the simultaneous stimulation (Fig 5C). We examined the effects of the SSRI fluvoxamine additionally, and found that fluvoxamine suppressed the levels of GTP-binding Rac (Fig 5D), p38 MAPK phosphorylation (Fig 5E) and HSP27 (Ser-78) phosphorylation (Fig 5F), increased by the simultaneous stimulation with the combination of collagen and CXCL12, as well as tramadol.

### Effects of morphine or reboxetine on the phosphorylation of p38 MAPK in human platelets induced by the simultaneous stimulation with collagen and CXCL12

**Fig 6:**
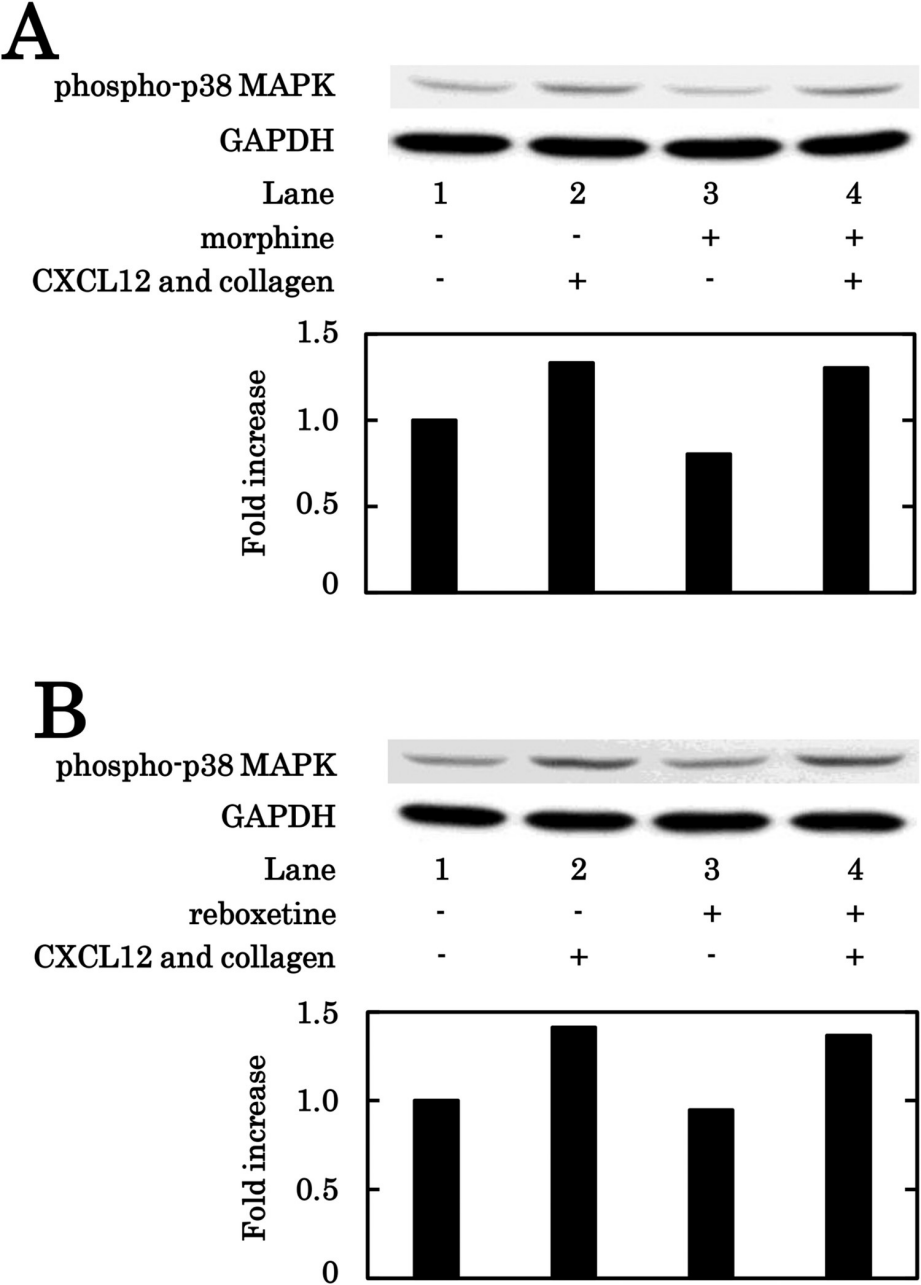
Effects of morphine or reboxetine on the phosphorylation of p38 MAPK in human platelets induced by simultaneous stimulation with collagen and CXCL12. PRP was pretreated with 200 μM of morphine (A), 100 nM of reboxetine (B) or vehicle for 3 min, and then simultaneously stimulated by 0.2 μg/ml (A) or 0.4 μg/ml (B) of collagen and 10 ng/ml of CXCL12 for 5 min. The reaction was terminated by the addition of ice-cold EDTA (10 mM) solution, and the levels of phosphorylated p38 MAPK in the cell lysate of platelets were determined by the Western blot analysis using anti-phospho-p38 MAPK antibodies or anti-GAPDH antibodies. The champion data of 3 times independent experiments are shown.

We also examined the effect of morphine on the p38 MAPK phosphorylation induced by the subthreshold levels of collagen and CXCL12 combination in human platelets. Morphine (200 μM) hardly affected the p38 MAPK phosphorylation (Fig 6A). We further examined the effect of reboxetine on the p38 MAPK phosphorylation, and found that reboxetine (100 nM) had little effect on the phosphorylation (Fig 6B).

## Discussion

In the present study, we investigated the effect of tramadol on the platelet activation induced by the combination of collagen and CXCL12 in their subthreshold levels to platelet aggregation. We demonstrated here that tramadol, which is pharmacologically a weak MOR agonist acting also as SNRI [3], suppressed platelet aggregation stimulated synergistically by the combination. In order to reveal the mechanism underlying the tramadol effect, we first examined the effect of a SNRI duloxetine [28] on the platelet aggregation stimulated synergistically by the combination of collagen and CXCL12, and found that the SNRI also suppressed the aggregation. We next examined the effects of SSRIs fluvoxamine and sertraline [4] and a noradrenaline reuptake inhibitor reboxetine [30] on the platelet aggregation stimulated by the combination. As results, we found that the SSRIs enabled to decrease the platelet aggregation, whereas the noradrenaline reuptake inhibitor failed to affect the aggregation. It is likely that the inhibition of not adrenaline reuptake but serotonin reuptake among tramadol actions may be involved in the suppression of the platelet aggregation stimulated by the combination of subthreshold collagen and CXCL12. We confirmed that the platelet aggregation was hardly affected by the MOR agonist morphine. Therefore, it seems likely that tramadol suppresses the platelet aggregation stimulated by the combination of subthreshold collagen and CXCL12 via the action to inhibit serotonin reuptake through SERT. In addition, we found that tramadol as well as fluvoxamine truly inhibited the secretion of PDGF-AB, the release of sCD40L and that of phosphorylated HSP27 induced by the synergistic stimulation of human platelets. It is likely that the inhibition of serotonin reuptake through SERT suppresses the platelet activation synergistically stimulated by the combination of subthreshold collagen and CXCL12.

Regarding the reuptake of serotonin via SERT in platelet, serotonylation, transamidation of cytoplasmic serotonin to small GTPases including Ras, Rho and Rac is considered to paticipate in platelet activation [9, 13, 37]. It is recognized that prenylation is required for the activation of small GTPases, and that farnesyltransferase for Ras and geranylgeranyltransferase for Rho and Rac catalyze the responsive prenylation [31]. We found here that not the FTase inhibitor III [32] but the geranylgeranyltransferase inhibitor GGTI-286 [31] suppressed the platelet aggregation synergistically stimulated by the combination of subthreshold collagen and CXCL12. It is likely that not Ras but Rho and/or Rac may be involved in the platelet aggregation. We then investigated whether or not the Rho kinase inhibitors fasudil and Y27632 [35] affect the platelet activation stimulated synergistically by the combination of collagen and CXCL12. We found that neither fasudil nor Y27632 affect the platelet aggregation, the secretion of PDGF-AB, and the release of sCD40L and phosphorylated HSP27 stimulated by the combination. As we confirmed here that these Rho kinase inhibitors truly suppressed the phosphorylation of cofilin induced by the synergistic stimulation of the combination, it is unlikely that the activated Rho may function in the regulation of platelet activation. We next examined the effect of NSC23766, the selective Rac-GEF interaction inhibitor [36], on the platelet activation stimulated synergistically by the combination of collagen and CXCL12, and found that NSC23766 suppressed not only the platelet aggregation but also the PDGF secretion, the sCD40L release and the phosphorylated HSP27 release induced by the synergistic stimulation by the combination, which truly increased the levels of GTP-binding Rac. Therefore, it is most likely that Rac is involved in the platelet activation induced by the synergistic effect of collagen and CXCL12.

**Fig 7:**
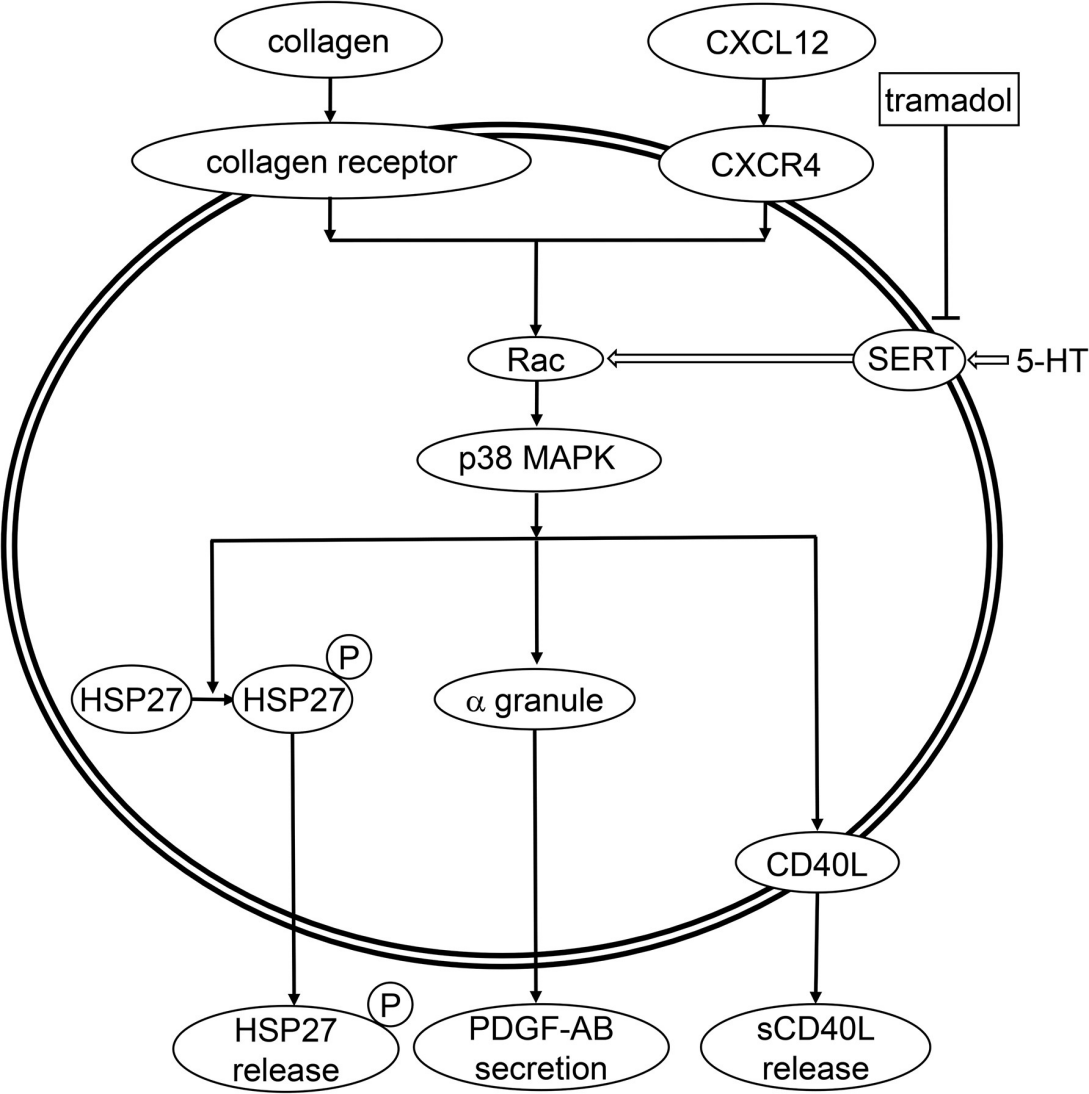
Diagram of the potential mechanism underlying the role of SERT in the platelet activation by simultaneous stimulation with subthreshold collagen and CXCL12. 5-HT, 5-hydroxy-tryptamine; SERT, serotonin transporter; MAPK, mitogen-activated protein kinase; HSP, heat shock protein; P, phosphorylation; PDGF-AB, platelet-derived growth factor-AB; CD40L, CD40 ligand; sCD40L, soluble CD40 ligand.

In the proceeding study [26], we reported that the simultaneous stimulation with the combination of subthreshold collagen and CXCL12 induces an activation of p38 MAPK and subsequent HSP27 phosphorylation, the former for both the PDGF-AB secretion and the sCD40L release and the latter for the release of phosphorylated HSP27. On the basis of these findings, we examined the effect of NSC23766 on the phosphorylation of p38 MAPK and HSP27 induced by the simultaneous stimulation of the combination. We showed clearly that NSC23766 truly suppressed the phosphorylation of p38 MAPK and HSP27 stimulated by the combination, suggesting that Rac probably regulates the activation of p38 MAPK and subsequent phosphorylation of HSP27 at the upstream in the activating platelets. We further examined the effect of tramadol on the levels of GTP-binding Rac upregulated by the simultaneous stimulation with the combination of subthreshold collagen and CXCL12, and found that the levels were decreased by tramadol. The phosphorylation of p38 MAPK and HSP27 induced by the combination was also inhibited by tramadol, suggesting that the tramadol-inhibited Rac serotonylation may decrease the p38 MAPK activation and subsequent HSP27 phosphorylation in the platelets stimulated synergistically by the combination. We also demonstrated that the SSRI fluvoxamine also decreased the levels of GTP-binding Rac, p38 MAPK phosphorylation and HSP27 phosphorylation induced by the simultaneous stimulation of the combination. Moreover, we confirmed that neither morphine nor reboxetine affected the phosphorylation of p38 MAPK simultaneously stimulated by the combination. Therefore, it is most likely that the SERT inhibition-caused decrease of serotonylation reduces the Rac activation, resulting in the suppression of p38 MAPK activation and subsequent HSP27 phosphorylation in the platelets stimulated synergistically by the combination. To take these findings into account, it is most likely that the inhibition of serotonin reuptake through SERT among tramadol actions and decrease of Rac activity at the upstream of p38 MAPK suppress the platelet activation synergistically stimulated by the combination of subthreshold collagen and CXCL12. The potential underlying mechanism of the tramadol action onto the human platelet activation synergistically stimulated by the subthreshold combination of collagen and CXCL12 is summarized in Fig 7.

Tramadol is broadly used as an analgesic for both acute and chronic pain including the neuropathic pain caused by diabetic neuropathy [5]. Treatment with tramadol has been actually associated with an increased risk of bleeding peptic ulcer [38]. On the other hand, it is evident that SSRI intake is a risk for bleeding complication associated with surgery [4]. In the guideline for interventional spine and pain procedures in patients on antiplatelet and anticoagulant medications, withdrawal of SSRI and SNRI is recommended before performing the procedure in patients with high bleeding risk [28]. From our present findings which were limited in the activation of platelets stimulated by the combination of collagen and CXCL12 in their subthreshold levels, tramadol should be handled equally to SSRI and SNRI as a highly risk factor of bleeding. As the peak concentration of tramadol in clinical use is recognized about 3 μM [27], the concentration of tramadol used in the present study was much greater than the therapeutic range, indicating that the concentration to reduce platelet activity would not be achieved therapeutically. However, it might be considerably essential to propose pharmacological new finding of tramadol from these ex-vivo experiments, which could be involved in unexpected clinical setting. Further investigations, in addition to using other agonists such as ADP, thrombin, adrenalin or arachidonic acid, quantifying the reduction in the extracellular concentration of serotonin with HPLC following the addition of serotonin in platelets, and quantifying serotonin on platelets supernatant in the presence or absence of tramadol, would be required to prove tramadol interference to SERT, leading to hemorrhagic complications.

In conclusion, our results suggest that tramadol negatively regulates the combination of subthreshold collagen and CXCL12-induced platelet activation via Rac upstream of p38 MAPK.

## Methods

### Materials

Collagen was purchased from Takeda Austria GmbH (Linz, Austria). Recombinant CXCL12, reboxetine mesylate, PDGF-AB enzyme-linked immunosorbent assay (ELISA) kit and sCD40L ELISA kit were purchased from R&D systems (Minneapolis, MN). Tramadol hydrochloride (tramadol), duloxetine hydrochloride (duloxetine) and fasudil were obtained from Sigma-Aldrich Co. (St. Louis, MO). Morphine hydrochloride was obtained from Takeda Pharmaceutical Co., Ltd. (Osaka, Japan). Fluvoxamine maleate, sertraline hydrochloride and NSC23766 were obtained from Tocris Bioscience (Bristol, UK). FTase inhibitor III, GGTI-286 and Y27632 were obtained from Calbiochem-Novabiochem (LaJolla, CA). Phospho-specific cofilin antibodies and phospho-specific p38 MAPK antibodies were obtained from Cell Signaling, Inc. (Beverly, MA). GAPDH antibody was obtained from Santa Cruz Biotechnology (SantaCruz, CA). Phospho-specific HSP27 (Ser-78) antibodies and phosphorylated-HSP27 (Ser-78) ELISA kit were purchased from Enzo Life Sciences, Inc. (Farmingdale, NY). A Rac1 Activation Assay Kit was obtained from Millipore Co. (Billerica, MA). Other materials and chemicals were obtained from commercial sources. Tramadol, FTase inhibitor III and GGTI-286 were dissolved in dimethyl sulfoxide ant other reagents were dissolved in water solution. The maximum concentration of dimethyl sulfoxide was 0.5%, which did not affect platelet aggregation, the protein detection using Western blotting or ELISA for PDGF-AB, sCD40L and phosphorylated-HSP27.

### Preparation of platelets

Human blood was collected from healthy volunteers without any medication under 50 years old and added into 1/10 volume of 3.8% sodium citrate immediately. Blood samples from healthy volunteers were collected without bed rest before work in a state of morning fasting. Blood samples from patients were collected after 30 minutes of bed rest without fasting. Platelet-rich plasma (PRP) was prepared by the centrifugation at 155 × *g* at room temperature for 12 min. Platelet-poor plasma (PPP) was obtained from the residual samples by the centrifugation at 1,400 × *g* at room temperature for 5 min. The Ethics Committee of Gifu University Graduate School of Medicine (Gifu, Japan) approved this study (2020–032). All methods were performed in accordance with relevant guidelines, regulations and the Declaration of Helsinki. Written informed consent was collected from all participants. Patients with chronic pain due to neuropathic pain were enrolled in the study and patients under antiplatelet medication were excluded.

### Platelet aggregation

Platelet aggregation was measured by using PA-200 aggregometer (Kowa Co. Ltd., Tokyo, Japan), which analyze the size of platelet aggregates based on particle counted by laser scattering methods (small; 9–25 μm, medium; 25–50 μm and large; 50–70 μm). Preincubation of PRP was at 37°C for 1 min with a stirring speed at 800 rpm. If indicated, PRP was pretreated with tramadol, morphine, duloxetine, reboxetine, fluvoxamine, sertraline, FTase inhibitor III, GGTI-286, fasudil, Y27632 or NSC23766 for 3 min, and then stimulated by 10 ng/ml of CXCL12 and subthreshold doses of collagen varied between 0.05 and 0.45 μg/ml. In all experiments, the dose of collagen was individually adjusted to subthreshold dose which cause a % transmittance of 10%-30% by the aggregometer. The platelet aggregation was monitored for 4 min. The percentage of transmittance of the isolated platelets was recorded as 0%, and that of the PPP was recorded as 100%. The data about transmittance, small aggregates, medium aggregates and large aggregates were expressed as black line, blue line, green line and red line, respectively, which were automatically presented by the aggregometer.

### Protein preparation after stimulation

Platelet aggregation was terminated by adding an ice-cold EDTA (10 mM) after the stimulation with CXCL12 and collagen. The mixture was centrifuged at 10,000 × *g* for 2 min at 4°C. The supernatant was collected for ELISA and stored at -80°C. The pellet was washed twice with phosphate-buffered saline (PBS) and then lysed by boiling in a lysis buffer 62.5 mM Tris-HCl, pH 6.8, 2% sodium dodecyl sulfate (SDS), 50 mM dithiothreitol and 10% glycerol for a Western blotting analysis [26].

### Western blotting

Western blot analysis was performed as described previously [39]. Briefly, SDS-polyacrylamide gel electrophoresis (PAGE) was performed as described by Laemmli in 10% or 12.5% polyacrylamide gel [40]. The proteins in the gel were transferred onto a PVDF membrane and blocked with 5% fat-free dry milk in PBS containing 0.1% Tween 20 (PBS-T; 10 mM Na_2_HPO_4_, 1.8 mM KH_2_PO_4_, pH 7.4, 137 mM NaCl, 2.7 mM KCl, 0.1% Tween 20) for 2 h, then incubated with the indicated primary antibodies. Peroxidase-labeled anti-rabbit IgG antibodies were used as secondary antibodies. The primary and secondary antibodies were diluted to optimal concentrations with 5% fat-free dry milk in PBS-T. The peroxidase activity on the PVDF membrane was visualized on X-ray film using an ECL Western blotting detection system (GE Healthcare, Buckinghamshire, UK) as described in the manufacturer’s protocol. A densitometric analysis was performed using a scanner and imaging software program (Image J version 1.50; NIH, Bethesda, MD). The levels of phosphorylation were calculated as follows: the background-subtracted intensity of each signal was normalized to the respective intensity of GAPDH and plotted as the fold increase compared with that of the control cells.

### ELISA for PDGF-AB, sCD40L and phosphorylated-HSP27

The levels of PDGF-AB, sCD40L and phosphorylated-HSP27 (Ser-78) in the supernatant of the conditioned mixture after platelet aggregation were determined using ELISA kits for PDGF-AB, sCD40L and phosphorylated-HSP27 (Ser-78) respectively in accordance with the manufacturer’s instructions [26].

### Analysis of Rac activation

The pellet obtained by platelet stimulation was washed twice with tris-buffered saline (TBS) and then lysed by sonication in Mg^2+^ lysis/wash buffer (MLB). GTP-binding Rac was immunoprecipitated using a Rac1 Activation Assay Kit as described in the manufacturer’s instruction manual. The immunoprecipitated GTP-binding Rac and pre-immunoprecipitated lysates, that is total Rac, were subjected to Western blot analysis using antibodies against Rac [41].

### Statistics

Statistical analyses were carried out using SPSS 15.0 (SPSS, Chicago, USA). The comparison of the values was done using Kruskal-Wallis ANOVA followed by Mann Whitney U test. A probability of <5% was considered to be statistically significant. The data were presented as the mean ± SEM.

## Supporting information

S1 Table Effects of tramadol, duloxetine, fluvoxamine, sertraline, morphine or reboxetine on the combination of collagen and CXCL12-indued platelet aggregation. (PDF)

S2 Table Effects of FTase inhibitor III, GGTI-286, fasudil, Y27632 or NSC23766 on the combination of collagen and CXCL12-indued platelet aggregation. (PDF)

S1 Raw images Original uncropped and unadjusted images of Figs 3C–3E, 4A, 4F, 4G, 5 and 6. (PDF)
