# Supplementary material for: Tramadol regulates the activation of human platelets via Rac but not Rho/Rho-kinase
Source: PLoS One. 2023 Jan 13;18(1):e0279011. doi: 10.1371/journal.pone.0279011 (PMC9838859; doi:10.1371/journal.pone.0279011)
Supplement: S1 Table — (PDF) [file pone.0279011.s001.pdf]

Supple. 1. Effects of tramadol, duloxetine, fluvoxamine, sertraline, morphine or reboxetine on the combination of collagen and CXCL12-indued platelet aggregation.

agent	dose (μM)	Transmittance (%)	Size of aggregation Large (%)	Size of aggregation Medium (%)	Size of aggregation Small (%)
tramadol	0	73.7 ± 4.3	56.3 ± 4.1	17.7 ± 1.5	26.0 ± 2.9
	100	53.3 ± 5.2*	32.0 ± 6.7*	24.3 ± 0.9*	44.0 ± 6.1*
	200	34.0 ± 1.5*	12.3 ± 3.4*	20.7 ± 2.3	67.0 ± 5.7*
	300	26.3 ± 1.2*	6.0 ± 1.0*	15.7 ± 0.3	78.3 ± 1.2*
duloxetine	0	74.7 ± 0.9	48.7 ± 3.8	19.0 ± 0.6	32.0 ± 3.8
	10	61.0 ± 1.5*	43.3 ± 3.2	21.7 ± 0.6	37.0 ± 0.6
	20	40.3 ± 3.8*	16.0 ± 3.8*	21.7 ± 1.9	62.3 ± 5.5*
	30	20.7 ± 3.8*	7.0 ± 1.5*	11.3 ± 2.6*	81.3 ± 4.3*
fluvoxamine	0	80.3 ± 3.5	58.3 ± 3.8	16.0 ± 0.6	25.7 ± 3.3
	10	75.3 ± 5.2	45.3 ± 1.8*	19.7 ± 1.2*	35.0 ± 1.0*
	20	56.3 ± 4.7*	25.3 ± 2.3*	24.0 ± 0.0*	50.7 ± 2.0*
	30	26.3 ± 6.6*	6.7 ± 3.2*	12.0 ± 2.0	81.3 ± 5.1*
sertraline	0	81.7 ± 5.5	47.0 ± 8.4	18.7 ± 1.8	34.0 ± 6.9
	10	54.3 ± 2.3*	23.3 ± 3.4*	22.3 ± 1.8	54.7 ± 4.8
	20	45.7 ± 9.0*	18.3 ± 9.6	17.7 ± 4.1	64.0 ± 13.4
	30	26.3 ± 1.2*	6.0 ± 1.0*	15.7 ± 0.3	78.3 ± 1.2*
morphine	0	80.0 ± 2.1	52.3 ± 1.8	17.7 ± 0.3	30.3 ± 1.9
	100	81.7 ± 5.5	48.7 ± 2.2	19.0 ± 0.6	32.0 ± 1.5
	200	83.0 ± 5.6	45.0 ± 0.6	18.7 ± 1.3	35.0 ± 0.6
	300	85.3 ± 5.2	51.0 ± 3.6	17.3 ± 0.3	32.3 ± 3.4
reboxetine	0	80.7 ± 6.8	49.3 ± 3.0	18.3 ± 0.9	32.0 ± 2.5
	30	76.7 ± 6.4	48.0 ± 5.0	19.0 ± 1.0	33.3 ± 3.7
	70	77.3 ± 4.7	48.0 ± 3.6	20.7 ± 0.7	31.0 ± 3.0
	100	72.0 ± 6.4	48.7 ± 7.6	17.0 ± 1.5	34.7 ± 6.4

PRP was pretreated with various doses of tramadol, duloxetine, fluvoxamine, sertraline, morphine or reboxetine for 3 min, and then simultaneously stimulated by collagen (0.075-0.45 μg/ml) and 10 ng/ml of CXCL12 for 5 min. The results analyzed with the aggregometer for the transmittance and the ratio of the platelet aggregates size in large, medium and small, are summarized. Each value represents the mean ± SEM of 3 healthy donors. *$p<0.05$, compared to the value of control in each.
