# Supplementary material for: Tramadol regulates the activation of human platelets via Rac but not Rho/Rho-kinase
Source: PLoS One. 2023 Jan 13;18(1):e0279011. doi: 10.1371/journal.pone.0279011 (PMC9838859; doi:10.1371/journal.pone.0279011)
Supplement: S2 Table — (PDF) [file pone.0279011.s002.pdf]

Supple. 2. Effects of FTase inhibitor III, GGTI-286, fasudil, Y27632 or NSC23766 on the combination of collagen and CXCL12-indued platelet aggregation.

inhibitor	dose (μM)	Transmittance (%)	Size of aggregation Large (%)	Medium (%)	Small (%)
FTase inhibitor III	0	80.7 ± 4.1	53.7 ± 4.4	21.7 ± 0.3	24.7 ± 4.7
	0.3	74.7 ± 3.2	50.7 ± 5.8	22.0 ± 0.0	27.3 ± 5.8
	1.0	75.0 ± 1.7	46.0 ± 2.3	25.7 ± 2.2	28.3 ± 3.0
	3.0	74.7 ± 3.0	49.0 ± 1.0	23.3 ± 3.3	27.7 ± 4.3
GGTI-286	0	82.3 ± 6.2	45.3 ± 9.1	30.7 ± 8.2	24.0 ± 6.2
	10	57.7 ± 7.0*	27.7 ± 11.0	47.3 ± 16.7	25.0 ± 7.8
	30	36.3 ± 4.6*	9.7 ± 6.2*	53.3 ± 19.6	37.0 ± 20.4
	50	24.0 ± 4.0*	3.0 ± 1.7*	62.3 ± 25.2	34.7 ± 24.9
fasudil	0	82.7 ± 4.3	43.0 ± 11.0	18.7 ± 2.0	38.7 ± 9.0
	10	78.7 ± 3.8	43.0 ± 11.5	17.0 ± 1.5	39.7 ± 10.3
	20	82.0 ± 4.6	39.3 ± 7.3	19.3 ± 0.3	41.3 ± 7.1
	30	79.3 ± 5.4	43.7 ± 9.9	16.3 ± 0.9	40.3 ± 8.8
Y27632	0	79.0 ± 1.0	40.3 ± 6.2	18.7 ± 0.7	41.0 ± 5.5
	10	81.0 ± 0.6	40.0 ± 5.0	18.0 ± 0.6	41.7 ± 4.8
	20	83.7 ± 1.9	40.7 ± 3.5	18.7 ± 0.3	40.7 ± 3.7
	30	81.7 ± 4.1	45.7 ± 4.9	16.3 ± 0.3	38.0 ± 4.5
NSC23766	0	78.7 ± 7.5	51.3 ± 4.2	19.0 ± 2.0	30.0 ± 2.2
	1.0	50.7 ± 8.2	26.3 ± 9.8	22.7 ± 1.7	51.0 ± 9.5
	2.0	26.3 ± 7.8*	9.3 ± 4.4*	15.7 ± 3.8	75.0 ± 8.1*
	3.0	17.0 ± 4.0*	6.7 ± 2.3*	10.7 ± 3.2	82.7 ± 5.4*

PRP was pretreated with various doses of FTase inhibitor III, GGTI-286, fasudil, Y27632 or NSC23766 for 3 min, and then simultaneously stimulated by collagen (0.075-0.45 μg/ml) and 10 ng/ml of CXCL12 for 5 min. The results analyzed with the aggregometer for the transmittance and the ratio of the platelet aggregates size in large, medium and small, are summarized. Each value represents the mean ± SEM of 3 healthy donors. **p*<0.05, compared to the value of control in each.
