# Supplementary material for: Tramadol regulates the activation of human platelets via Rac but not Rho/Rho-kinase
Source: PLoS One. 2023 Jan 13;18(1):e0279011. doi: 10.1371/journal.pone.0279011 (PMC9838859; doi:10.1371/journal.pone.0279011)
Supplement: S1 Raw images — (PDF) [file pone.0279011.s003.pdf]

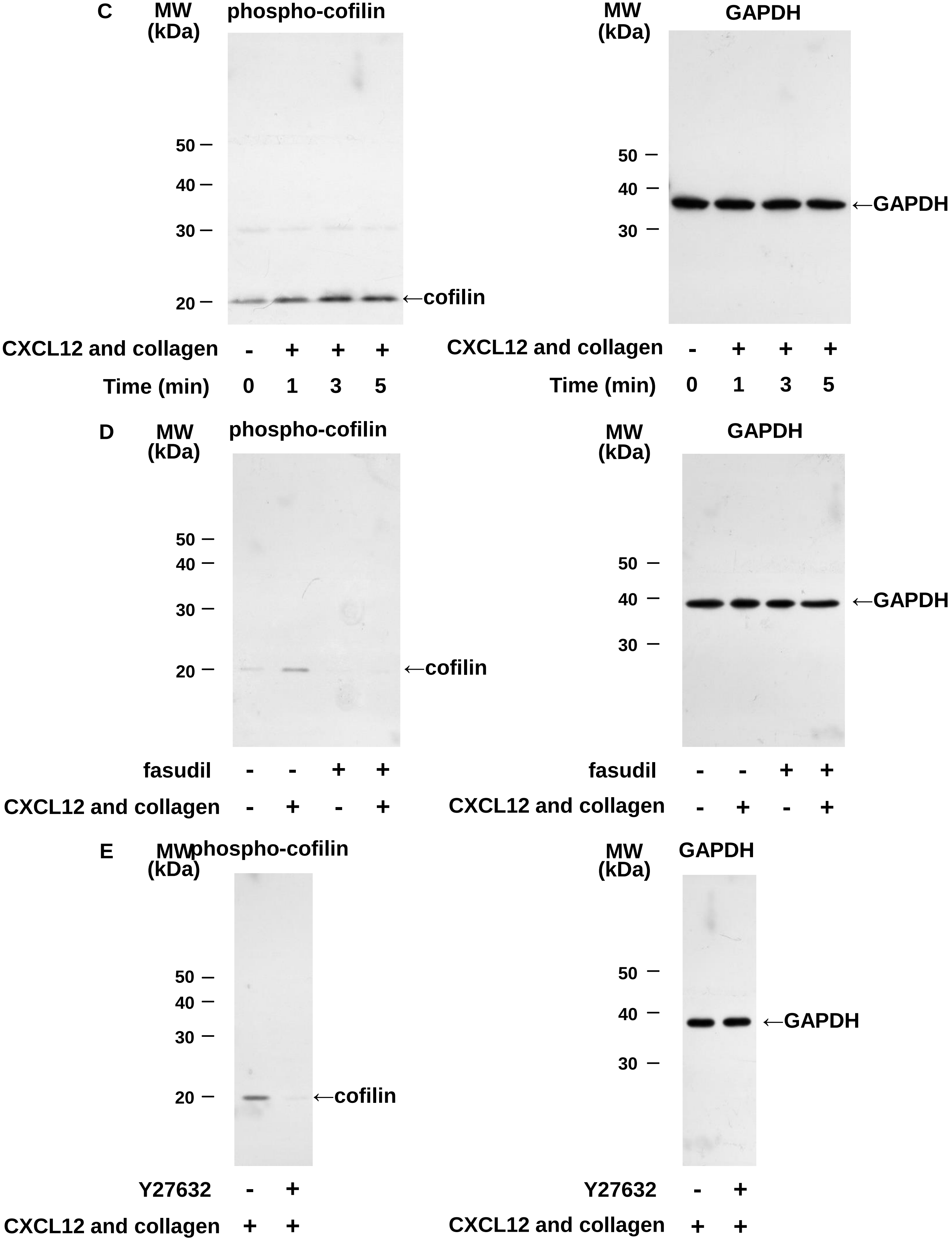

Supple. 3. Full-length blot images for Figure 3.

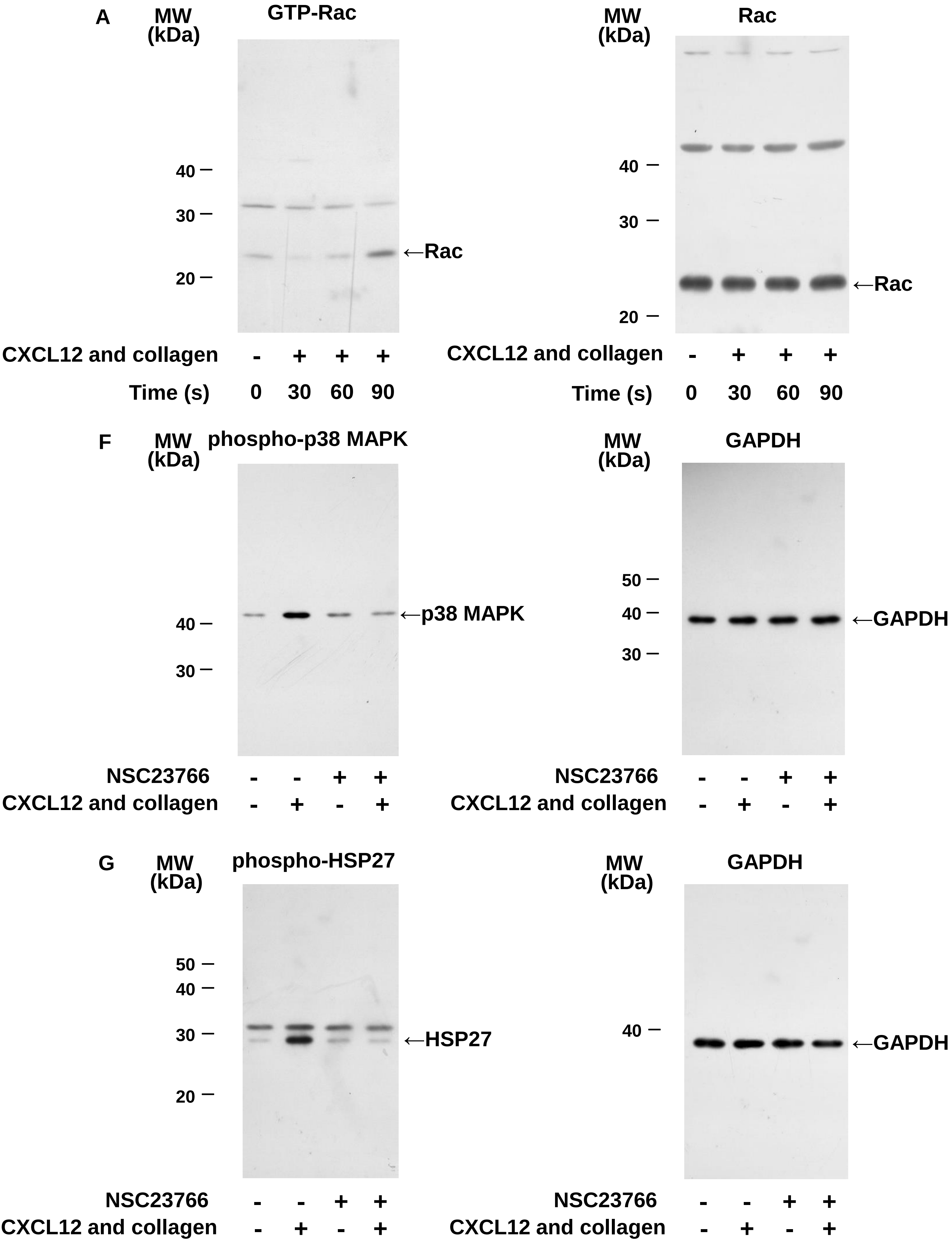

Supple. 4. Full-length blot images for Figure 4.

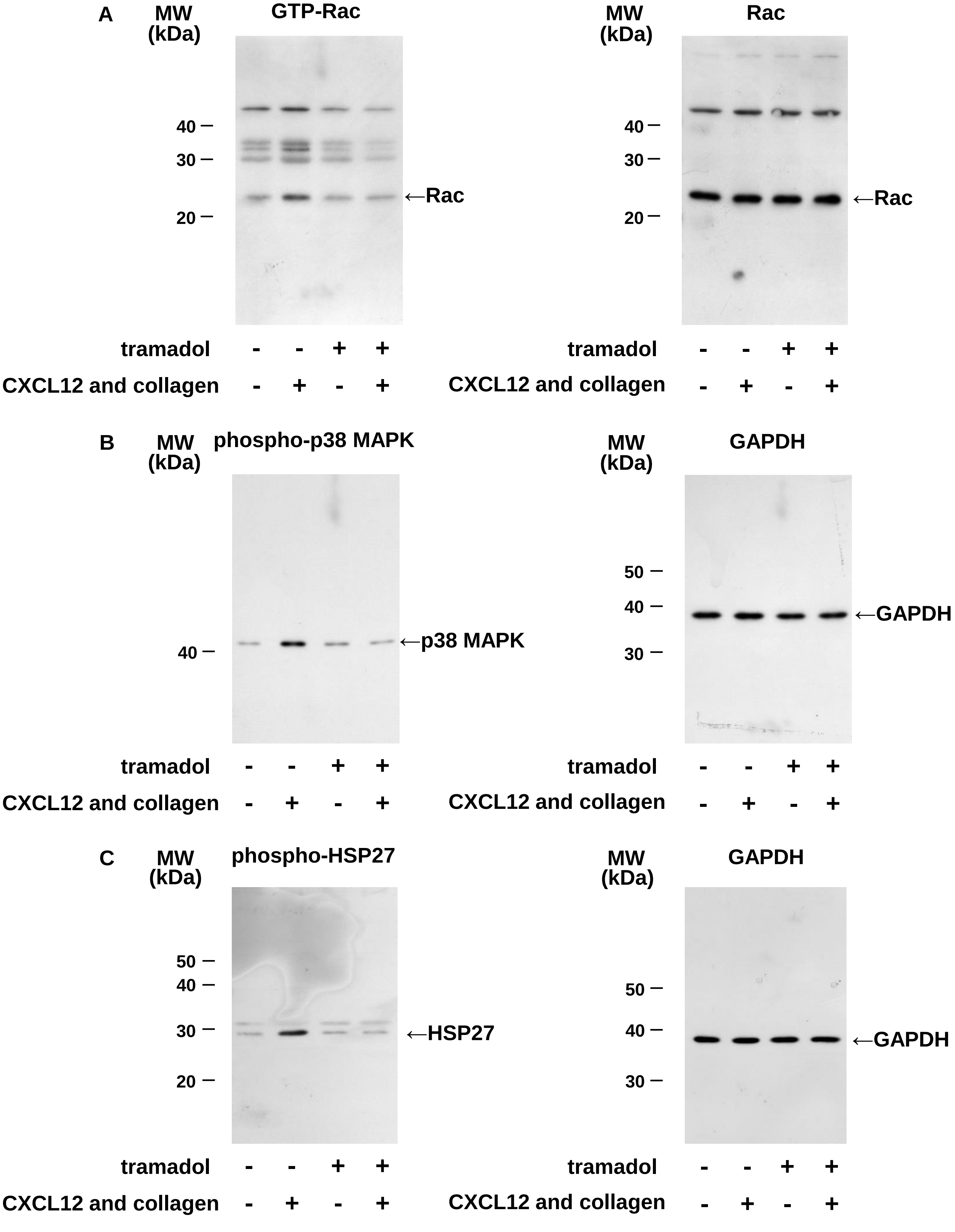

Supple. 5-1. Full-length blot images for Figure 5A-5C.

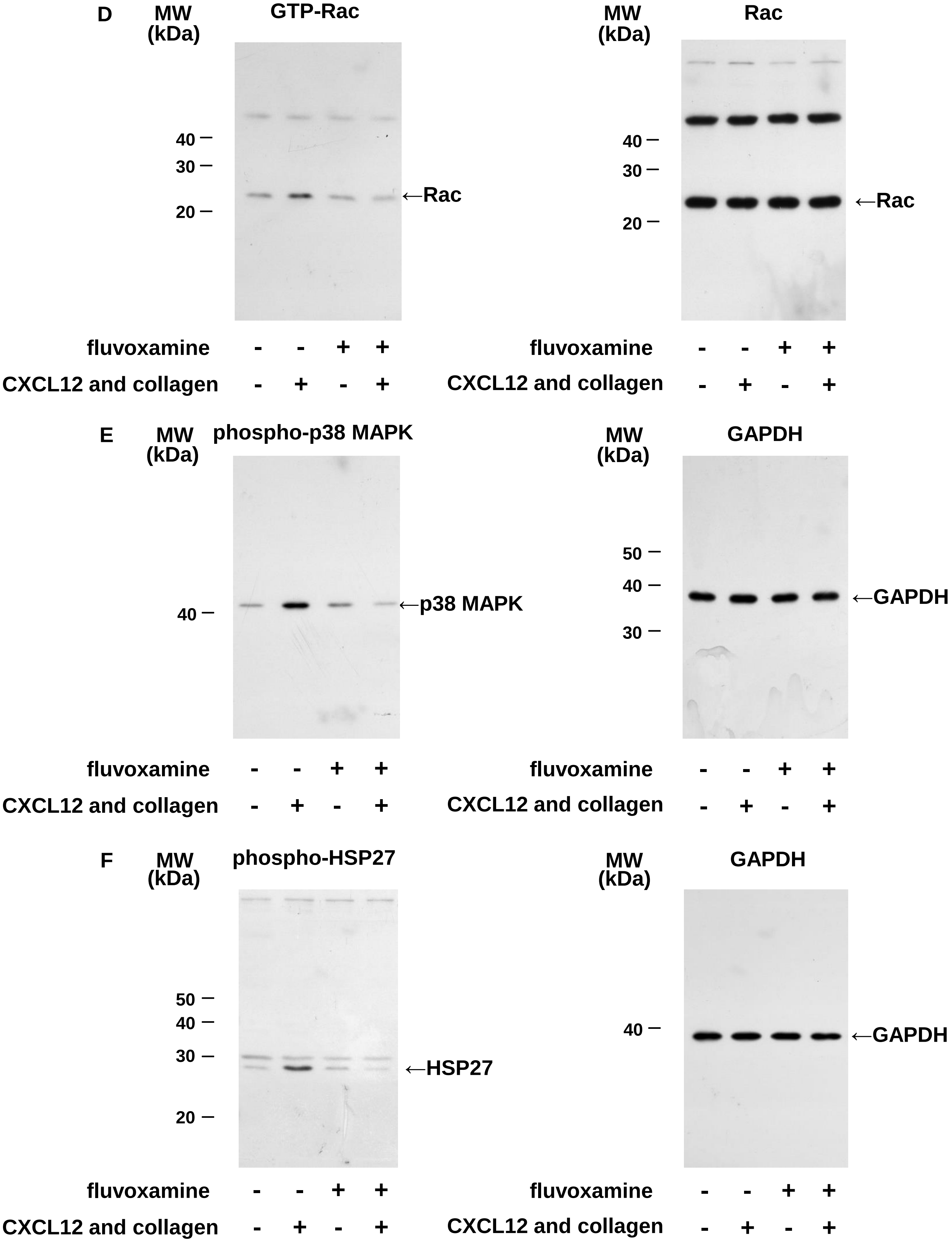

Supple. 5-2 . Full-length blot images for Figure 5D-5F.

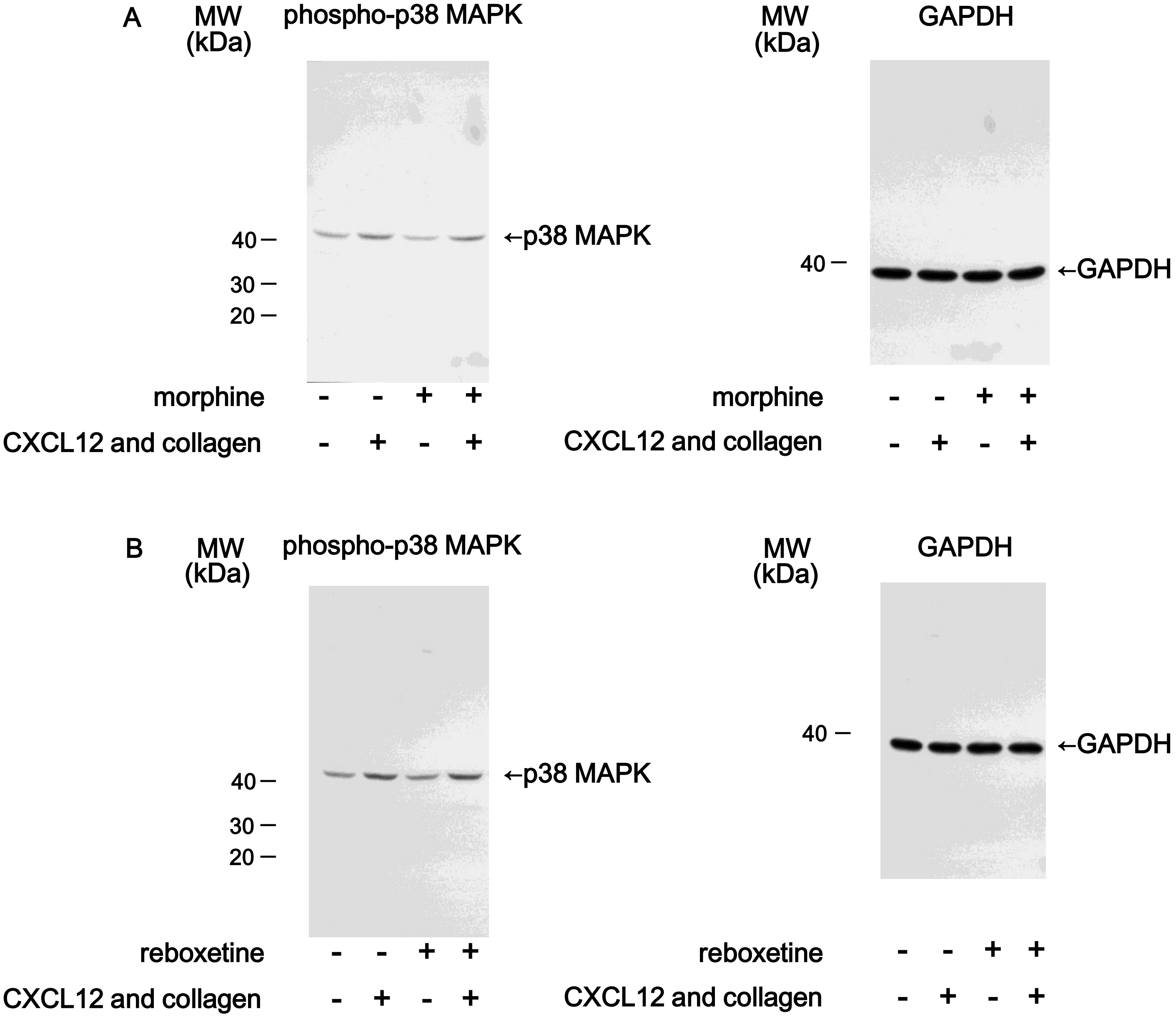

Supple. 6 . Full-length blot images for Figure 6A-6B.
